# English Lexical Analysis System of Machine Translation Based on Simple Recurrent Neural Network

**DOI:** 10.1155/2022/9702112

**Published:** 2022-06-16

**Authors:** Jingyan Zhu

**Affiliations:** School of Foreign Languages, Henan University of Animal Husbandry and Economy, Zhengzhou, Henan 450046, China

## Abstract

With the increasing communication between countries, machine translation has become an important way of communication between ethnic groups in different language systems. In order to further improve the coverage mechanism and vocabulary translation quality of the machine translation model of neural network, this paper will study the machine translation English lexical analysis system based on simple recurrent neural network. In order to improve the effect of word alignment, marking and attention mechanism are introduced into the English lexical analysis model. The experimental results show that the tagging of named entity words in the system can reduce the problem of unregistered words. Combined with the attention mechanism, it can significantly improve the effect of word alignment and label recall. It not only improves the controllability of translation but also has a positive impact on the quality of translation and its application effect in specific scenes. The evaluation of translation quality indicators shows that the system can effectively improve the accuracy and quality of Chinese-English translation.

## 1. Introduction

At present, long short-term memory (LSTM) and recurrent neural network (RNN) are widely used in machine translation. The model is good at modeling natural language, transforming sentences of any length into floating-point vectors of specific dimensions, and “remembering” the more important words in the sentences for a long time. The model well solves the problem of sentence vectorization in natural language and is of great significance for using computer to process natural language so that the language processing by computer no longer stays at the simple level of literal matching but further goes deep into the level of semantic understanding.

Language exchange is an important way of cultural exchange among countries all over the world. Due to the differences in language and culture between countries, there are some obstacles in the communication between ethnic groups with different language systems. With the advantage of fast translation speed, machine translation has become an important way of communication between different languages. Especially in recent years, the development of computer technology has provided technical support for machine translation, and machine translation equipment that can carry out real-time translation has been applied in many fields [1]. At present, the machine translation method based on neural network has become an important development direction of machine translation. However, due to the limitation of the capacity of translation vocabulary and coverage mechanism, there are some problems in machine translation based on neural network, such as unregistered words, overtranslation, or missing translation [2]. Therefore, the optimization and improvement of machine translation based on neural network have become the focus of machine translation research. In language translation, the mutual translation between English and different languages is the most common and widely used, and the language rules of different language systems are different [3]. Words are the basic elements of modern English expression, and each word may correspond to multiple words, and the semantics of each sentence can be described correctly only by dividing the words contained in it into a single meaning. The results of English word segmentation have a very important impact on the translation effect of machine translation model. Therefore, this paper proposes the research of machine translation English lexical segmentation system based on simple recurrent neural network, which can improve the word alignment effect and translation quality of the system by tagging unlisted words and attention tailoring and regularization.

This paper will study the machine translation English lexical analysis system based on simple recurrent neural network. The study is divided into five parts. The first part expounds the background content of language rules of different language systems. The second part analyzes the development and current situation of machine translation research. The third part constructs a machine translation English vocabulary analysis model based on simple recurrent neural network. A simple recurrent neural network is described. This paper analyzes the English vocabulary analysis model of machine translation based on simple recurrent neural network. The fourth part analyzes the experimental results of machine translation English vocabulary analysis system based on simple recurrent neural network. Finally, the full text is summarized. The results show that this study can significantly improve the effect of word alignment and label recall. It not only improves the controllability of translation but also has a positive impact on the quality of translation and its application effect in specific scenes.

## 2. Development and Current Situation of Machine Translation Research

Machine translation is a way to realize the conversion between different natural languages through computer technology. It was first realized through dictionary matching, but this way is only a rigid conversion between words, ignoring the language characteristics of different languages [4]. To solve this problem, scholars continue to accumulate language rules and knowledge of different languages and introduce language expert knowledge rules to optimize translation results on the basis of dictionary translation [5]. However, when the number of relevant rules in this kind of machine translation system increases to a certain number, it is difficult to improve the system performance through rule increase [6]. After that, some scholars put forward a way to translate the sentences produced by the new source language with the help of relevant principles according to the existing translation experience and knowledge. The language database automatically summarized in this way and the rule design can eliminate the problem of ambiguity, and the translation result is better than the rule-based machine translation [7, 8]. Based on this, some scholars have proposed a method to locate continuous and discontinuous boundary matching according to the alignment type of fragments in the translated language sentence [9]. With the development of computer technology and data statistics technology, the idea of machine translation based on statistical technology has been put forward; that is, the translation task is transformed into the probability of any two sentences translating each other between corpora. The key point is to find a suitable way to match the source sentence with the target slogan sentence with the maximum probability [10]. Some studies have listed the availability of machine translation language modeling tools, parser databases, and evaluation indicators. The classification of MTS, evaluation methods, and platforms is based on a clear set of criteria. The survey article explores new research approaches, which will help to develop high-quality MTS [11]. Some fish species and other important biological information are artificially inferred. Large-scale visual inspection is reliable and highly automated. Some experts combined convolutional neural network (CNN) with transfer learning to apply it to a new prediction of fish (wild/cultured) [12]. However, the focus of this translation method is to divide sentences into words, ignoring the influence of the positional relationship between words. With the development of statistical machine translation, it has gradually become the mainstream of machine translation, but there are still some problems to be solved in practical application, such as linear indivisibility, difficult design features, and insufficient utilization of nonlocal context [13]. By 2012, deep learning came into people's vision and the stage of explosive development began. Machine translation based on neural network came into being [14]. Neural network machine translation can alleviate the problems of statistical machine translation to a great extent and gradually show better translation performance. It has become the core technology of many commercial online machine translation systems [15]. It is generally composed of encoder and decoder structures, and their network structures can be different [16]. At the same time, in the process of translation, neural network machine translation is mainly carried out according to the mapping relationship between the two languages, which greatly simplifies the complexity of the translation model [17]. At present, the development of neural network machine translation has encountered a bottleneck. Problems such as unregistered words, overtranslation, and exposure deviation restrict its performance improvement. Solving these problems has become the main direction of neural network machine translation model research [18].

## 3. Construction of English Lexical Analysis Model of Machine Translation Based on Simple Recurrent Neural Network

### 3.1. Simple Recurrent Neural Networks

**Figure 1:**
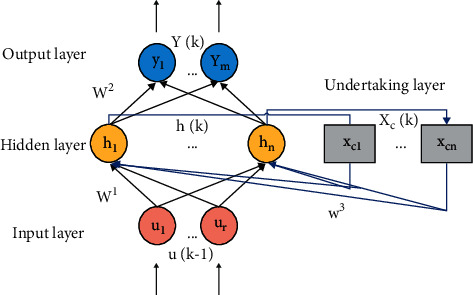
Schematic diagram of Elman network structure in simple recurrent neural network.

The development and application of artificial neural network and deep learning theory have solved many problems and bad phenomena in statistical machine translation and improved the performance of machine translation [19]. Feedforward neural network plays an important role in various project classifications with its unique advantages, but its function also has some limitations. However, there are some limitations; for example, the performance is not as good as machine learning under small data samples, the model is relatively complex, and the process cannot be explained [20]. In contrast, the feedback neural network has better stability in learning and memory performance. Among them, the simple recursive neural network not only has fast convergence speed and high accuracy but also can retain the historical input information and take it as the network output while analyzing the logical information sequence and practical relationship in the information [21]. The simple recurrent neural network structure has the same input layer, hidden layer, and output layer as the feedforward neural network, as well as the receiving layer connected with the hidden layer, and each neuron in the receiving layer has and only has one corresponding hidden layer neuron. Its main function is delay feedback, that is, to memorize and store the data information output by the corresponding hidden layer neuron at the previous time, and delay feedback back to the hidden layer neuron [22]. Each time a new input is input, the LSTM will first decide which memories to forget according to the new input and the output of the previous time; the input and the output of the previous step will be integrated into a separate vector and then through the sigmoid neural layer and finally point-to-point multiplication on the unit state. Because the sigmoid function will compress any input to the interval of [0, 1], we can intuitively get the working principle of this gate; if a component of the integrated vector becomes 0 after passing through the sigmoid layer, it is obvious that the corresponding component of the unit state will also become 0 after bit multiplication. In other words, “forgetting” the information on this component, if a component is 1 after passing through the sigmoid layer, the cell state will “keep a complete memory.” Different sigmoid output will bring different information memory and forgetting. In this way, LSTM can remember important information for a long time, and the memory can be adjusted dynamically with the input. This can increase the dynamic memory performance of simple recurrent neural network and maintain strong historical data sensitivity.  Figure 1 shows the structure of Elman network in simple recurrent neural network.

The mathematical model is shown in the three following formulas:

(1) $$\begin{array}{c} y\left(k\right)=g\left(w^{2}h\left(k\right)+b_{y}\right), \end{array}$$

(2) $$\begin{array}{c} h\left(k\right)=f\left(w^{1}u\left(k-1\right)+w^{3}x_{c}\left(k\right)+b_{h}\right), \end{array}$$

(3) $$\begin{array}{c} x_{c}\left(k\right)=\alpha \cdot x_{c}\left(k-1\right)+h\left(k-1\right). \end{array}$$

In the above formulas, the iterative time step is expressed as *k*, the input vector and output vector are expressed as *u*=[*u*_1_, *u*_2_,…, *u*_*r*_] and *y*=[*y*_1_, *y*_2_,…, *y*_*m*_], respectively, the vector of the hidden layer is expressed as *h*=[*h*_1_, *h*_2_,…*h*_*n*_], and the output vector of the receiving layer is expressed as *x*_*c*_=[*x*_*c*1_, *x*_*c*2_,…, *x*_*cn*_]. The connection weight matrix is expressed as *w*^1^, *w*^2^, *w*^3^, and the first two can be modified and updated, and the latter is a fixed value. The threshold matrix is expressed as *b*_*y*_ and *b*_*h*_. The activation function is expressed as *g*(•), *f*(•).

Simple recurrent neural network extracts the dynamic characteristics of input and output parameters and determines the stable network parameters in the learning process. Its learning algorithm selects error correction learning; that is, when there is an error beyond the acceptable range between the actual value and the expected value output by the output layer, it will distribute the error signal layer by layer to the neurons of each layer by means of back-propagation, and, on this basis, update and correct the weight and threshold matrix of neurons of each layer. Let the actual output of the simple recurrent neural network be expressed as *y*(*k*) and let the expected output value be expressed as $\hat{y}\left(k\right)$. The error function is shown in the following formula:

(4) $$\begin{array}{c} E\left(W\right)\left(k\right)=\frac{1}{2}{\left(\hat{y}\left(k\right)-y\left(k\right)\right)}^{T}\left(\hat{y}\left(k\right)-y\left(k\right)\right). \end{array}$$

The goal of learning and training simple recurrent neural network is to obtain the minimum weight matrix that meets the *E*(*W*^*∗*^)=min*E*(*W*) condition. Therefore, this problem can be transformed into an optimization problem, which can be solved by learning BP algorithm. The weight matrix update formulas are shown in the two following equations:

(5) $$\begin{array}{c} W=W+\Delta W, \end{array}$$

(6) $$\begin{array}{c} \Delta W=-\eta \frac{\partial E}{\partial W}. \end{array}$$

The error function is expressed as *E* and the learning rate is expressed as *η*.

The calculation of weight correction between hidden layer and output layer is shown in the following formula:

(7) $$\begin{array}{c} \Delta w_{ij}^{2}=\eta \left({\hat{y}}_{i}\left(k\right)-y_{i}\left(k\right)\right)g_{i}^{\prime }\left(•\right)h_{j}\left(k\right),\;i=1,2,\ldots ,m;j=1,2,\ldots ,n. \end{array}$$

The calculation of skirt paper correction between input layer and hidden layer is shown in the following formula:

(8) $$\begin{array}{c} \Delta w_{jq}^{1}=\eta \sum_{i=1}^{m}\left({\hat{y}}_{i}\left(k\right)-y_{i}\left(k\right)\right)g_{i}^{\prime }\left(•\right)w_{ij}^{2}f_{i}^{\prime }\left(•\right)u_{q}\left(k-1\right),\;j=1,2,\ldots ,n;q=1,2,\ldots ,r. \end{array}$$

The calculation of the weight correction amount before the receiving layer and the hidden layer is shown in the following formula:

(9) $$\begin{array}{c} \Delta w_{jl}^{3}=\eta \sum_{i=1}^{m}\left\{\left({\hat{y}}_{i}\left(k\right)-y_{i}\left(k\right)\right)g_{i}^{\prime }\left(•\right)w_{ij}^{2}\right\}\frac{\partial h_{i}\left(k\right)}{\partial w_{jl}^{3}},\\ \frac{\partial h_{i}\left(k\right)}{\partial w_{jl}^{3}}=f_{i}^{\prime }\left(•\right)h_{l}\left(k-1\right)+\alpha \frac{\partial h_{i}\left(k-1\right)}{\partial w_{jl}^{3}},\;j=1,2,\text{…},n;l=1,2,\text{…},n. \end{array}$$

The activation function is shown in the two following formulas:

(10) $$\begin{array}{c} f\left(\sigma \right)=\frac{1}{1+e^{-\sigma }},\;0<f\left(\sigma \right)<1, \end{array}$$

(11) $$\begin{array}{c} f\left(\sigma \right)=\frac{e^{\sigma }-e^{-\sigma }}{e^{\sigma }+e^{-\sigma }}=2\left(\frac{1}{1+e^{-2\sigma }}\right)-1,\;-1<f\left(\sigma \right)<1. \end{array}$$

### 3.2. English Lexical Analysis Model of Machine Translation Based on Simple Recurrent Neural Network

**Figure 2:**
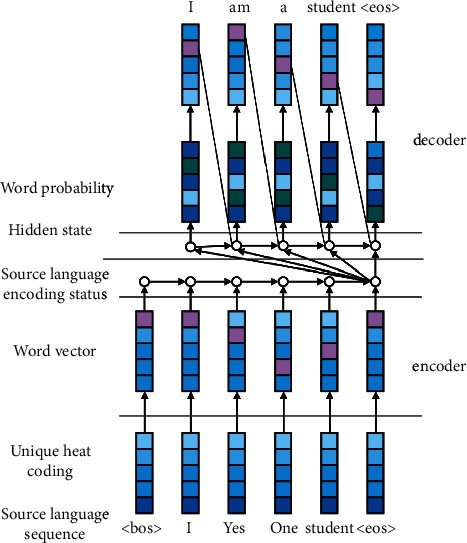
Encoder-decoder framework of machine translation English lexical analysis system based on simple recurrent neural network.

The basic idea of end-to-end neural machine translation is to directly map the source language text to the target language text using neural network. Different from statistical machine translation, there are no more manually designed late structures such as word alignment, phrase segmentation, and syntax tree. There is no need for artificial design features; end-to-end neural machine translation can directly realize the conversion of natural language text only using a nonlinear neural network. During English translation, there is complete alignment between the translation source and the target, resulting in unequal sentence length or mismatching of words between the source and the target. Therefore, it is necessary to combine the structure of simple recurrent neural network model with end-to-end translation thinking.  Figure 2 shows the encoder-decoder framework of machine translation English lexical analysis system based on simple recurrent neural network.

The encoder collects and obtains the corresponding data information of the input source word sequence by the coding process in the time dimension; the decoder gradually translates the time series and outputs the results. The process is shown in the two following formulas:

(12) $$\begin{array}{c} s_{t}=f_{1}\left(s_{t-1},o_{t-1},c\right), \end{array}$$

(13) $$\begin{array}{c} o_{t}=f_{2}\left(s_{t},o_{t-1},c\right). \end{array}$$

Finally, take the maximum dimension of the output vector soft max as the current decoding result, as shown in the following formula:

(14) $$\begin{array}{c} y_{t}=\arg\max_{d}\left\{\text{soft}\max\left(W_{y}o_{t}+b_{y}\right)\right., \end{array}$$

Machine translation is based on English lexical information. It pays attention to the cognition and processing of the basic units contained in the text. Choosing the appropriate way of word segmentation and marking can help the machine translation English word segmentation system based on simple recurrent neural network improve its performance. In the process of machine translation, the particularity and coincidence of the meaning of named entities and rare words will affect the system performance. This paper deals with this problem through the word based on attention mechanism. First, take the last step result of the decoder unit and the RNN transmission implicit state as the input value, and the output value is calculated as shown in the following formula:

(15) $$\begin{array}{c} o_{t}=\text{LSMT}\left(s_{t-1},y_{t-1}\right). \end{array}$$

The output value is expressed as *o*_*t*_, which is matched and normalized with the implicit state correlation in the coding stage, as shown in the two following formulas :

(16) $$\begin{array}{c} \text{score}\left(o_{t},h_{j}\right)=v_{a}^{T}\tanh\left(W_{a}\left[o_{t},h_{j}\right]\right), \end{array}$$

(17) $$\begin{array}{c} \alpha_{t,j}=\frac{\exp\left(\text{score}\right.\left(o_{t},h_{j}\right)}{\sum_{k=1}^{T}\exp\left(\text{score}\left(o_{t},h_{k}\right)\right)}. \end{array}$$

The implicit state of each step is *h*_*t*_.

The resulting output implicit vector is shown in the following formula:

(18) $$\begin{array}{c} c_{t}=\sum_{j=1}^{T}\alpha_{t,j}*h_{j}. \end{array}$$

The output hidden vector is expressed as *c*_*t*_ and the length of neural network is expressed as *T*.

Due to the relatively perfect extraction method of named entity words, the coding method of unregistered words can be expanded with its tagging characteristics, and the diversity of coding methods also makes different decoding methods more feasible. The application of word alignment concept not only makes the entity label output by the target end correspond to the entity word or label at the source end but also combines the attention mechanism to normalize the score for all source words, which not only increases the robustness of word alignment results but also further improves the accuracy in the process of understanding code replacement. On this basis, if we strengthen the translation effect of named entity tags, we can not only improve the recall rate of named entity tags in decoding but also have a very important impact on the accuracy of tag translation. The optimization of attention mechanism is the key to improve the alignment probability of entity tags. Therefore, based on the idea of coverage mechanism, this paper proposes a clipping and regularization training method for attention score.

Coverage mechanism has been applied in statistical machine translation, which can avoid obvious errors, repeated translation, or floor translation in the process of translation. In machine translation based on simple recurrent neural network, coverage mechanism can help it obtain the most reasonable word alignment method, improve the ability of input sentence coverage and word alignment, and reduce the error rate of named entity translation. According to the idea of coverage, the machine translation English word segmentation system based on simple recurrent neural network should score the source words that have been paid attention to and have not been paid attention to in the historical steps in the decoding process, that is, reduce the attention of the words that have been paid attention to and shift the attention to the words that have not been translated, so as to dynamically adjust the attention of different decoding steps.

After all decoding processes are completed, the variables that record the attention history will have certain semantic information due to their distribution mode, which may affect the law of the overall attention canvas. Therefore, the attention can be closer to the preset distribution mode through the attention distribution regularization process. Compared with the coverage mechanism, the combination of attention clipping mechanism and regularization does not damage the translation effect. The regularization method discards the realization of the weight of the source word of implicit variable learning and selects the information extraction ability of coding modeling method to realize the convergence in the reasonable expression interval. This method has higher controllability. Therefore, this paper selects the combination of attention clipping mechanism and regularization.

## 4. Experimental Result

### 4.1. Analyze the System Performance Test Results

Considering the experimental effect and actual situation, this paper selects the common Chinese-English translation for testing and selects the Chinese-English bilingual alignment corpus that provides a high-quality open-source dataset for corresponding training. The length threshold in the training is set to 100, and 10000 sentence pairs of the corpus are randomly selected as the verification set to observe the convergence degree and generalization state of the parameters, so as to prevent the system from overfitting. It is also the comparison object of the experimental results. This paper mainly investigates the recall rate and attention distribution regression of unregistered words, in which the threshold of unregistered words is set to 50.

**Figure 3:**
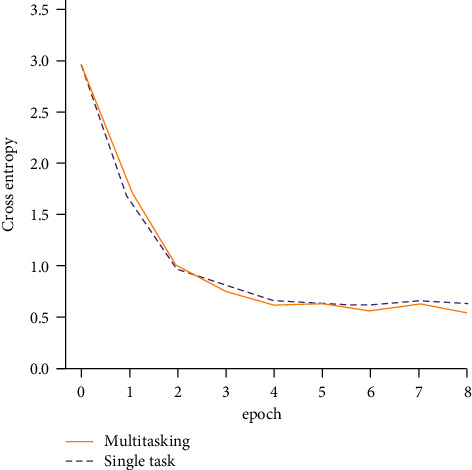
The cross entropy curve of the prediction and target results of the entity label in the verification set.

The coding preprocessing of the system adopts named entity labeling, which also enables the named entity location task of the target language to be trained synchronously. In the experiment, the convergence of the named entity location task will be tested, and the change of the prediction accuracy of the named entity label in the training process will be analyzed. The experiment will be expressed by the prediction of the entity label in the verification set and the cross entropy of the target result. The results are shown in Figure 3. It can be seen from the results in the figure that the overall development trend of the cross entropy value of multiple tasks is a downward trend with the increase of the number of training cycles, and its downward trend is similar to the convergence law of conventional single task training, which shows that the named entity calibration and positioning task in the system can still carry out normal training.

**Figure 4:**
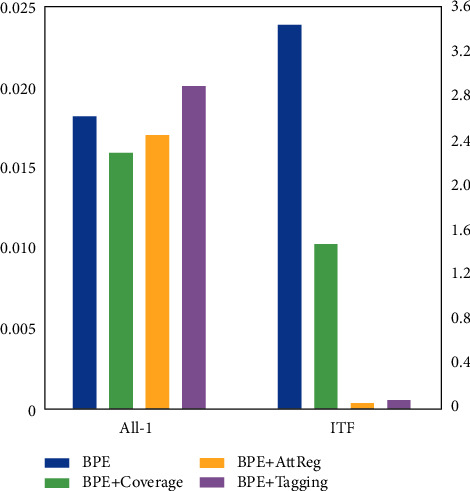
Variance of attention records and target distribution in different machine translation methods.

The regularization effect of attention mechanism has a key impact on the translation effect of entity tags, and its convergence effect and distribution state reflect the rationality of regularization. Therefore, this paper records the overall attention of different machine translation methods on the verification set and also records the mean square error of their target distribution. The results are shown in Figure 4. According to the results in the figure, RNN search model and coverage model are test comparison models. The coverage model adopts different ways to realize the effect of attention history on decoding, but it does not adjust the loss function accordingly. In the simple uniform distribution (all-1) state, the results of all translation models in the experiment are almost the same, and all have minimal variance. This shows that attention sharing strategy is the optimal choice of machine translation model based on neural network. In the state of inverse word frequency exponential distribution (ITF) with high complexity, the differences between the translation models in the experiment are obvious. The convergence of RNN search model and coverage model has little effect, but the way of combining attention clipping and regularization in this paper shows good adaptability; that is, this way has a positive effect and great impact on the machine translation English word segmentation system based on simple recurrent neural network. On this basis, combined with tagging, the influence of changing the coding method on the regularization effect of attention can be ignored.

**Figure 5:**
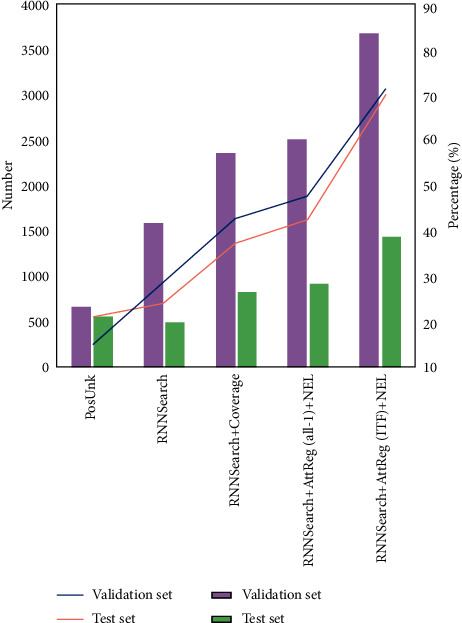
Comparison results of label recall quantity and ratio under single tag by different machine translation methods.

The UNK substitution method in the system has a high demand for the recall rate of unregistered words in the decoding process. The higher the recall rate, the more unregistered words that can be reasonably translated by this method. In order to better verify the effect of the system, the unlisted words are marked by the traditional word segmentation method, as shown in Figure 5. The results show that the alignment strategy of the first method is based on the position relationship between the source end and the target end, but its performance is poor. Although the second method introduces the word alignment translation model, its performance and stability are poor. The third way is to combine the coverage mechanism in the original basic model. Although it significantly improves the effect of word alignment, it is less than half in the recall rate. Because the method proposed in this paper has completed the positioning training for unlisted words, its recall rate has been significantly improved again, and its effect in all-1 and ITF is significantly different. The regularization effect in the all-1 state is relatively poor, and there is no obvious difference between the status of tags and common words in the regularization process, so the overall improvement effect is limited. In the ITF state, the system improves the tags with low word frequency and the weight of words in a wide range, which makes the word alignment performance of unregistered words more prominent under the attention mechanism, which is more than 20% higher than that in the all-1 state.

**Figure 6:**
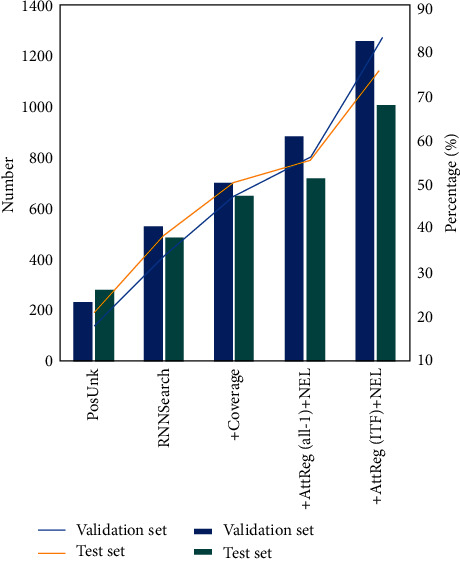
Comparison results of label recall quantity and ratio under entity labels by different machine translation methods.

Figure 6 shows another set of experimental results on the dataset that has been preprocessed after the system named entity tag optimization. After the system optimization, the processing of unregistered words can laugh out the UNK label and only label the location of the named entity of the unregistered words. It can be seen from the data in the figure that the new English word segmentation method proposed in this paper performs better in terms of recall rate. At the same time, tagging is more concentrated in the location of named entities, which improves its coverage. This shows that the introduction of this word segmentation method in the system can not only improve the translation performance of unlisted words but also have a good effect on the retrieval of tags. The increase of label recall rate increases the controllability of translation, and the translation effect and application effect of specific scenes are also improved.

To sum up, compared with other neural network machine translation and traditional machine translation, the machine translation English lexical system based on simple recurrent neural network has better performance and significantly improves the label recall rate, which can strengthen the effect of English translation and provide technical guarantee for the application of the system.

### 4.2. Evaluation Results of English Lexical Analysis System of Machine Translation Based on Simple Recurrent Neural Network

Blue quality evaluation index is an index to measure the quality of translation model. It is mainly a quality evaluation algorithm designed based on the cooccurrence frequency of words and phrases. Suppose that the candidate translation sentence is expressed as *c*, the reference translation sentence is expressed as *s*, and the cooccurrence frequency of the *kn* group is expressed as *h*_*n*,*k*_. The calculation formulas are shown as follows:

(19) $$\begin{array}{c} {\text{CP}}_{n,k}\left(c,s\right)=\frac{\min\left(h_{n,k}\left(c\right),h_{n,k}\left(s\right)\right)}{\sum_{i}h_{n,i}\left(c\right)}, \end{array}$$

(20) $$\begin{array}{c} {\text{CP}}_{n}\left(c,s\right)=\sum_{k}{\text{CP}}_{n,k}\left(c,s\right). \end{array}$$

In order to reduce the demand for unreasonable conditions, the length penalty factor is introduced, and its calculation formula is shown as follows:

(21) $$\begin{array}{c} \text{BP}\left(c,s\right)=\left\{\begin{array}{cc} 1, & \text{if }l_{c}>l_{s},\\ 1-\left(l_{s}/l_{c}\right) & \text{if }l_{c}\leq l_{s}. \end{array}\right. \end{array}$$

The length penalty factor is BP, the candidate sentence length is *l*_*c*_, and the reference sentence length is *l*_*s*_. The weighted value of the scoring index in the *Nn* meta phrase that completes the punishment is shown in the following formula:

(22) $$\begin{array}{c} {\text{BLEU}}_{N}\left(c,s\right)=\text{BP}\left(c,s\right)*\;\exp\left(\sum_{n=1}^{N}\omega_{n}\log\;{\text{CP}}_{n}\left(c,s\right)\right). \end{array}$$

**Figure 7:**
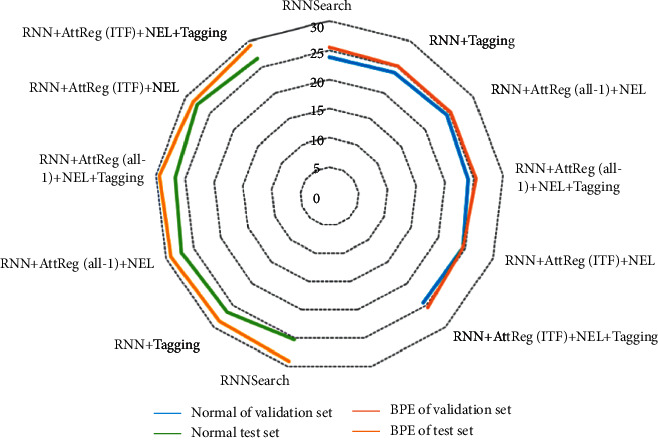
BLEU score of English-Chinese translation tasks with different machine translation methods.

As shown in Figure 7, the blue score results of translation tasks with different translation methods in the verification set and the test set are compared. Looking at the data in the picture as a whole, we can see that different English lexical ideas will have a great impact on the quality of the translation model. The optimization of lexical information can improve the performance of conventional word segmentation. Posunk shows obvious inadaptability in the language translation with significant differences between Chinese and English, which is reflected in the translation effect. In contrast, RNN search based on attention score performed better. Both coverage mechanism and attention regression mechanism are formed by introducing directional constraints on the basis of attention mechanism. Both of them can greatly improve the translation quality of the system; in particular, the index score of the method proposed in this paper is relatively high. In addition, by adding different set tags to mark the unregistered named entities, the evaluation indicators can be improved to a certain extent. Compared with the results obtained only through attention regularization, the complexity of entity tags will reduce the evaluation indicators. This shows that adding entity tags can optimize word alignment and word position, but it will also improve the training intensity of neural network and increase the task of capturing semantic information of different tags.

**Figure 8:**
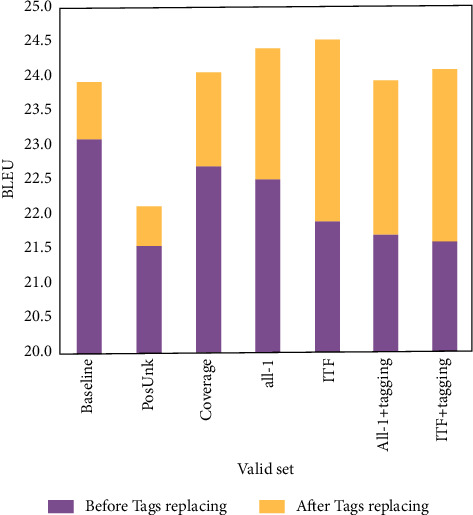
BLEU score improvement result of verification set in case of label replacement.

The increase of word alignment effect of the system has a great impact on the improvement of BLEU score; that is, the key influencing factor of machine translation quality is the effect of word alignment in translation results and translation labels. Therefore, in this paper, the results of the verification set of the conventional English lexical analysis method are counted separately in the label in the nonreplaced translation results and the score after replacement. The results are shown in Figure 8. According to the results, different optimization methods basically reduce the quality of machine translation to a certain extent before label replacement. After label replacement, the improvement of translation quality by label replacement makes up for the decline of this part, and the final performance of the system is improved. This shows that although the performance of machine translation English lexical analysis system based on simple recurrent neural network will be reduced by attention regularization, the improvement of word alignment performance has more impact on the increase of system translation quality than the negative impact of regularization, so that the overall effect and performance of the system remain stable when enhancing the translation effect of named entities. The direct impact of the regularization of attention mechanism on the quality of systematic translation is not clear.

**Figure 9:**
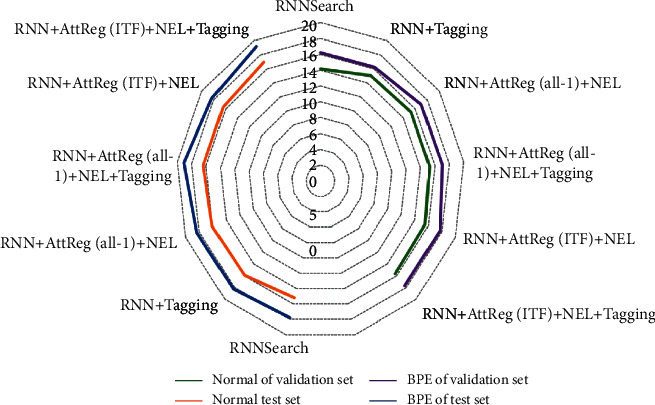
Comparison of BLEU scores of Chinese-English translation tasks completed by different translation methods under English lexical optimization.

For the translation effect test of different methods in the case of English lexical optimization, this paper chooses to evaluate the translation result indicators in the state of Chinese-English translation, and the results are shown in Figure 9. It can be seen from the figure that the performance of Chinese-English translation task in the relative relationship of results is similar to that of English-Chinese translation task, and the English lexical analysis method proposed in this paper can improve the effect of word segmentation and translation to a certain extent on the basis of attention mechanism. However, in the English-Chinese translation task, due to the differences between English Morphology and Chinese, the effect of systematic English lexical analysis method in English-Chinese translation is limited.

## 5. Conclusion

Not only can machine translation complete the translation task in a short time, but also its cost is relatively low, so it has been widely used in many fields. With the rise of neural network, machine translation has developed from statistical machine translation model to neural network machine translation model, which improves the translation effect. However, there are still some problems in machine translation based on neural network, such as overtranslation and the influence of unregistered words. Aiming at these problems, this paper proposes a machine translation English lexical analysis system based on simple recurrent neural network. Based on the machine translation model of simple recurrent neural network, the unregistered words in the system are marked, and the word alignment effect of the system is improved by combining attention clipping and regularization. The experimental results show that the machine translation English lexical analysis system based on simple recurrent neural network can complete its location and show good adaptability while naming and labeling entity words. Compared with other machine translation methods, it has obvious advantages in improving word alignment and recall. There are still some limitations in this paper. The research has not passed the real translation quality evaluation index. Although the standardization of attention mechanism in the system will reduce the translation effect, the positive impact of word alignment and tagging on translation effect cannot fully make up for this loss, which has limited promotion effect in English-Chinese translation tasks, and there are still some problems that need to be further optimized.

## Data Availability

The data used to support the findings of this study are available from the corresponding author upon request.
